# INGUINAL REPAIR VIA ROBOTIC ASSISTED TECHNIQUE: LITERATURE REVIEW

**DOI:** 10.1590/0102-672020180001e1408

**Published:** 2018-12-06

**Authors:** Eduardo Henrique PIROLLA, Gabriel Pavani PATRIOTA, Fernanda Junqueira Cesar PIROLLA, Felipe Piccarone Gonçalves RIBEIRO, Marina Guitton RODRIGUES, Layla Riva ISMAIL, Raquel Mezzalira RUANO

**Affiliations:** 1 Hospital Sírio-Libanês, São Paulo, SP, Brazil

**Keywords:** Inguinal hernia, Robotics, Laparoscopy, General surgery, Robotic surgical procedures, Hérnia inguinal, Robótica, Laparoscopia, Cirurgia geral, Procedimentos cirúrgicos robóticos

## Abstract

**Introduction:**

Inguinal hernia is one of the most frequent surgical diseases. Currently, with the advantages of minimally invasive surgery, new questions arise: what will be the best approach for correction of inguinal hernia? Is there real benefit to the robotic approach?

**Objective:**

To compile results of the published studies that used the robot-assisted technique in the repair of inguinal hernia, analyzing its limitations, complications and comparing it with those of the pre-existing techniques.

**Method:**

The review was performed from the Medline database with the following descriptors: (inguinal hernia repair OR hernioplasty OR hernia) AND (robot OR robotic OR robotic assisted) being retrieved 391 articles. After verification of the titles and abstracts, we identified eight series of cases congruent with the objectives of this review. Three reviewers participated in the extraction and selection of results.

**Results:**

Comparative studies showed an increase in surgical time in relation to the open and videolaparoscopic approach. The complications present similar rates with the other repair routes.

**Conclusion:**

This technique has been shown to be effective for the correction of inguinal hernia, but the benefits of using robotic surgery are unclear. So, there is a need for randomized studies comparing laparoscopic to robotic repair

## RESUMO

**Introdução:**

A hérnia inguinal é uma das doenças cirúrgicas mais frequentes. Atualmente, com as vantagens da cirurgia minimamente invasiva, novas questões surgem: qual será a melhor abordagem para correção de hérnia inguinal? Existe benefício real com a abordagem robótica?

**Objetivo:**

Compilar resultados dos estudos publicados que utilizaram a técnica robô-assistida no reparo da hérnia inguinal analisando suas limitações, complicações e comparando-a com as das técnicas pré-existentes.

**Método:**

A revisão foi realizada a partir da base de dados do Medline com os seguintes descritores: (inguinal hernia repair OR hernioplasty OR hernia) AND (robot OR robotic OR robotic assisted) sendo recuperados 391 artigos. Após verificação dos títulos e resumos, identificou-se oito séries de casos congruentes com os objetivos desta revisão. Três revisores participaram do processo de extração e seleção de resultados.

**Resultados:**

Nos estudos comparativos demonstrou-se aumento no tempo cirúrgico em relação à via aberta e videolaparoscópica. As complicações apresentam taxas similares com as outras vias de reparo.

**Conclusão:**

Esta técnica demonstrou-se efetiva para correção da hérnia inguinal, mas os benefícios da utilização da cirurgia robótica não estão claros. Para isso, há a necessidade de trabalhos randomizados que comparem o reparo laparoscópico ao robotizado.

## INTRODUCTION

Approximately 750,000 repairs of inguinal hernias are performed annually in the United States, and most American surgeons still prefer to perform the classic (open) repair technique^16^. Currently we have the presence of three validated and recommended techniques for correction of the inguinal hernia: open repair without a mesh (Shouldice technique), open repair with mesh (Lichtenstein technique - the most used) and laparoscopic technique, which involves the trans-abdominal pre-peritoneal (TAPP) and totally extra-peritoneal (PET) approaches^5^.

Several studies have demonstrated the benefits of inguinal repair through minimally invasive techniques, including less postoperative pain and early return to work and daily activities^2^. In addition, according to comparative studies^4^, laparoscopic repair also offers significant advantages in cases of bilateral inguinal hernias and recurrent inguinal hernias, since it makes detection easier and allows correction of the contralateral defect with minimal increase in surgical time^3^.

Robotic repair using the TAPP approach was first described by urological surgeons who performed such a procedure successfully during a robotic prostatectomy^7^ ^,^ ^16^. Given that 5-10% of patients undergoing this procedure have concomitant inguinal hernias, the robotic correction of both defects became common among these surgeons, allowing the observation of favorable outcomes in relation to exclusive radical prostatectomy with second-time repair of inguinal hernia^13^.

Current literature has eight case series on the use of the robot-assisted technique for inguinal hernia repair. In this way, we carry out this review in order to summarize the results.

## METHOD

The review was performed from the Medline database with the following descriptors: (inguinal hernia repair OR hernioplasty OR hernia) AND (robot OR robotic OR robotic assisted) being retrieved 391 articles. After evaluation of the titles and abstracts, eight series of cases congruent with the objectives of this review were identified. Three reviewers participated in the extraction and selection of results.

## RESULTS

### Surgical technique

The operative technique follows the concepts of the laparoscopic approach. The patient is positioned in dorsal decubitus and in Trendelenburg; first-generation cephalosporin antibiotic prophylaxis is performed. Trocars are inserted: one for the robot camera with diameter of 8-12 mm superiorly to the umbilical scar with angle of 0^º^ to 30^º^; another 12 mm inserted laterally in the mid-clavicular line at the level of the umbilical scar. The trocars should keep a distance of 15 cm between each one, in order to avoid difficulty in relation to the mobility of the robot’s arms^1^ ^,^ ^10^ ^,^ ^17^.

**FIGURE 1:**
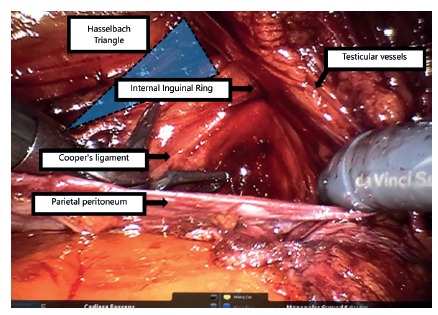
Representation of the Hesselbach triangle

Under the TAPP approach the incision in the peritoneum is curvilinear, above the hernia defect, between the medial umbilical ligament and the anterosuperior iliac spine^13^. Careful dissection of the pre-peritoneal fat can be performed with the use of electrocautery or ultrasonic scissors, in order to create peritoneal flaps/resections, being careful not to injure the inferior epigastric vessels^1^ ^,^ ^13^. These vessels laterally delimit the Hesselbach triangle, having, as its other limits, the inguinal ligament (inferiorly) and the border of the rectus abdominis (superiorly), represented in Figure 1.

**FIGURE 2:**
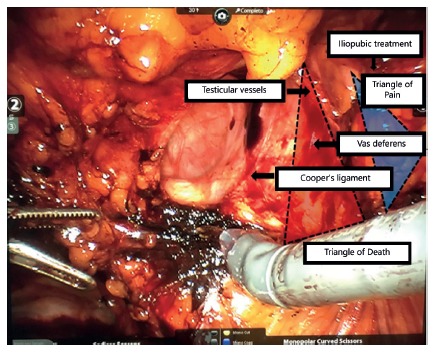
Representation of the triangles of death and pain

The peritoneal dissection delimits the pre-peritoneal space, medially confined by the pubic symphysis, laterally by the anterosuperior iliac spine, inferiorly by the pectineum or Cooper ligament, posteriorly by the testicular vessels and the posterolaterally retroperitoneal plane^10^. We identified at this level the Cooper’s ligament (laterally to the pubic symphysis), the Doom triangle (delimited medially by the vas deferens, laterally by the spermatic vessels and containing the iliac artery and vein). In the vicinity of the Doom triangle, there is another topography of great relevance called the pain triangle, delimited superiorly by the ileopubic tract and medially by the spermatic vessels. The two triangles are shown in Figure 2.

Once the triangles of Doom and pain have been identified, lateral dissection of the pre-peritoneal space can be initiated in order to separate the hernial sac to its extreme limits and thus allow the identification of the spermatic cord and the vas deferens that were spared^1^ ^,^ ^13^. In most cases, involved in the review, it was possible to reduce the hernial sac. In cases of this sac being very large, transection with ligation of its pedicle, without reduction of the distal portion, was an option at the discretion of each surgeon^10^ ^,^ ^13^. The importance of a 12 mm trocar is based on the easeness of placement the chosen mesh, besides the possibility of using staplers with larger diameters and special clamping clips for the cases of super obese patients.

The proximal peritoneal flap was obtained by suturing with an absorbable thread. The shape of the mesh fixation varied greatly in the studies, as will be described later. Fixation with absorbable stitches or stapling with absorbable surgical staples in the Cooper ligament was recommended medially and superiorly and two points against the pre-peritoneal fascia of the rectus abdominis, respecting the topographies of the mentioned triangles and the epigastric vessels.

In the inferior part of the mesh, no sutures or staples were used, adjusting it properly. The pre-peritoneal space is also closed with surgical staples. After the procedure, the robot is undocked and the umbilical portal is removed.

Regarding the mesh types used, the self-fixed one (Progrip®) was used in three studies^1^ ^,^ ^6^ ^,^ ^15^, with the fixation with silk stitches in one of them^13^. Two other studies have used polypropylene meshes^12^ ^,^ ^17^; in one of them the fixation was with fibrin glue (Tissel or Baxter)^17^. There was also the use of a self-adjusting polyester mesh with microstaples^7^ and mesh fixation with Prolene^®13^. Only one study did not specify the mesh type used^9^. 

### Studies characteristics 

In relation to the eight case series, seven were carried out in the United States and one in France. All the studies presented as a surgical approach the TAPP technique; however, they differed in the choice of mesh type and the technique for its fixation. Regarding the degree of experience of the surgeons, the studies were quite heterogeneous (Table 1).

**TABLE 1:** Characteristics of the studies

Author and year	Country	Robot	Surgeon experience
Eric J. Charles ^19^	USA	Da Vinci® Si Surgical System	Performed by two surgeons (previously having performed open and videolaparoscopic mode).
David S. Edelman, M.D ^12^	EUA	XI robot	Performed by only one surgeon
Ramachandra Kolachalam ^10^	EUA	Da Vinci® Surgical System	The seven surgeons evaluated had extensive experience with the open technique; three minimal prior laparoscopic experience; and four moderate to advanced laparoscopic experience.
Andrew Iraniha ^17^	EUA	Da Vinci® Si Surgical System	It does not specify if there was prior experience of the surgeon.
MassimoArcerito^1^	EUA	Da Vinci® Si Surgical System	Presents the first cases of hernia repair robot-assisted technique. It does not specify the number of surgeons involved.
Jose E. Escobar Dominguez ^13^	EUA	Da Vinci® Surgical System (Si or Xi depending on the surgeon's preference and systems viability)	Professionals with extensive experience in robotic surgery and previous experiences with the TEP approach in both laparoscopy and robotic surgery.
C. Engan ^6^	França	Da Vinci® Si Surgical System	It presents the first cases of correction of inguinal hernia by means of the technique robot-assisted by the surgical team. Only one surgeon was involved.
Jessica Gonzalez-Hernandez ^15^	EUA	Da Vinci® Si Surgical System	Compares inguinal repair via robotics performed by two surgeons with or without the participation of residents in the controls. Residents were previously qualified with online simulations and modules.

### Characteristics of patients

Considering the eight studies, there are a total of 747 patients, ranging in age from 16 to 96 years. The body mass index varied according to the selection of patients in each study from 24.34 to 34.2 kg/m² (Table 2).

**TABLE 2:** Characteristics of patients

	Patient	Age (years)	BMI (kg/m²)	Follow-up	Time for the 1st consultation after the operation
Eric J. Charles ^9^	69	52 (39-62 )	24.9 (22.9- 28.7 )	______	30 days
David S. Edelman, M.D ^12^	154	57 (21-85)	24.34 (19- 31.6)	16 weeks	2 weeks
Ramachandra Kolachalam ^10^	148	54,6 (±12.4)	34.2 (±4.9)¹	______	30 days
Jose E. Escobar Dominguez ^13^	78	55,1	27,6	______	7 days
MassimoArcerito^1^	Men 62 Women: 16	56 (25-96)	26 (±5.4)	12 months (±6)	2 weeks
C. Engan ^6^	Men: 30 Women: 4	49,3 (16-80)	26,5 (19.8-40.4)²	5,5 months (1-10)	2 weeks
Andrew Iraniha ^17^	82	52,86 (17-83)	26,44 (16.47-35.62)	12-36 months	2-6 weeks
Jessica Gonzalez-Hernandez ^15^	104	R³: 57,5 (±14,1) nR: 50,6 (±13,1)	R: 27,6 (±4,8) nR: 29,3 (±4,7)	______	R : 17 days nR : 21 days

¹BMI greater than 30 kg/m² in all patients; ²obese represented 15% of the sample (n=5); ³¹R=residents; nR=without residents’ participation

### Surgical time

Regarding surgical time, a great variability was observed, with an average time of 52 min up to 109.3 min (76-164) per hernia. Comparative analysis is difficult, since not all studies have specified operative time in relation to repairs of unilateral or bilateral hernias (Table 3).

**TABLE 3:** Surgical time of each study involved in minutes

	Mean operating time	Time for bilateral repair	Time for unilateral repair	Time for recurrent hernia repair
Eric J. Charles ^9^	105 (76-146)	Not specified	Not specified	Not specified
David S. Edelman, M.D ^12^	63.6 (25-140 )	Not specified	Not specified	Not specified
Ramachandra Kolachalam ^10^	87.9 (±35,6)	Not specified	Not specified	Not specified
Jose E. Escobar Dominguez ^13^	104,3 (±32,6)	107,8 (±28,26)	99,4 (±37.6)	Not specified
Massimo Arcerito^1^	52 (45-67)	Not specified	Not specified	Not specified
C. Engan ^6^	80.5 (45-135)	110 (84-135)	69 (45-128)	108 (67-135)
Andrew Iraniha ^17^	Not specified	98,57	Not specified	Not specified
Jessica Gonzalez-Hernandez ^15^	Not specified	R¹: 115.5 (±24,6) nR²: 109.3 (±55,4)	R: 73.2 ± 18.4 nR:67.3 ± 29.9	Not specified

²Obese represented 15% of the sample (n=5); ¹R=residents; nR=without residents’ participation

In a comparative analysis between the surgical time of the assisted or open robotic approaches, after adjusting for the interference variables, a significant value of p<0.001 was obtained in the study of Lawrence D'Amico et al.^10^ in the group submitted to the laparoscopic technique (Table 4). The adjusted variables for the correspondence were: age, gender, BMI, presence of concomitant procedure in the repair of inguinal hernias, primary hernias vs. recurrent, unilateral or bilateral repairs, presence of comorbidities, previous abdominal surgery and ASA classification. In the study of Eric Charles et al.^9^ the robotic modality also presented greater time when compared to videolaparoscopy and the open technique (p<0.001, Table 4).

**TABLE 4:** Comparative surgical time in the studies of D'Amico et al.^10^ and Charles et al.^9^

	Assisted robotic repair	Open technique	Laparoscopic technique	P
D'Amico et al.^10^				< 0,001
n	95	93	Not specified	
Total surgical time	82,9 min (±35,7)	51,5 (±20.9)	Not specified	
Charles et al.^9^				<0.001
n	69	191	241	
Total surgical time	105 (76-146)	71 (56-88) min	81(61-103)	

### Complications

In summary, the incidence of complications after assisted robotic inguinal repair was low. In the studies comparing it directly with the open technique, as in Lawrence D'Amico et al.^10^ the incidence of complications in robotic repair was 2.7% compared to 11.5% of traditional repair (p=0.005). As to the quality of the complications, there was a great deal of heterogeneity in the studies, being the seromas and hematomas in general more common.

It is worth mentioning the high incidence of hematomas in the study by Massimo Arcerito et al. (20%)^1^ which, according to the authors, is due to the high number of inguinoscrotal hernias among the selected patients. In almost all studies seromas did not require treatment; only in one case in the study of Jessica Gonzalez-Hernandez^15^ was outpatient aspiration. Escobar Dominguez et al.^13^ still mentioned two cases of chronic hematoma, defined by persistence for more than 30 days; the treatment was conservative in both cases.

The cases of urinary retention were not serious and in which the permanence of the Foley catheter was imperative, it did not exceed for more than a week^9^ ^,^ ^13^ ^,^ ^17^. Surgical site infections at the site of trocar insertion were not reported in all studies, but oral antibiotic prophylaxis was found to be adequate in those who did so.

Eric Charles et al.^9^ compared the robotic approach with the open and laparoscopic techniques, finding a higher rate of postoperative skin and soft tissue infection in patients submitted to the robotic technique that was associated with longer surgical time^9^. Four were cases of recurrence of the hernia. Andrew Iraniha et al.^17^ detailed that the recurrence of the right inguinal hernia occurred 20 weeks after the robotic repair and was treated by open path with mesh placement. On the other hand, Massimo Arcerito et al.^1^ reported medial recurrence to the placed tissue and made a re-approach via robotics for removal and re-placement of the prosthesis^1^. The hernia repair remained intact. Andrew Iraniha et al.^17^ reported a case of small bowel obstruction due to adherence to the remaining V-loc suture, which was resolved with laparoscopic adhesion lysis and no long-term sequelae were observed. Finally, in the study by Jessica Gonzalez-Hernandez^15^ there was a case that required conversion to open procedure due to the inability to obtain adequate pneumoperitoneum in a patient who had a history of previous abdominoplasty.

There was no report of a fatal outcome in any of the studies.

 In 2018 a study was published showing similar rates of postoperative complications between the resident group and the nonresident group^15^ with p-value without statistical significance. Among them: urinary retention - 11.1% vs. 2%; hematoma/edema - 18.5% vs. 10%; burning/numbness - 5.8% vs. 2.1% and infection - 0% in both groups. 

## DISCUSSION

In view of the advantages observed with laparoscopic inguinal repair over time, especially in cases of greater operative difficulty in the open route, and the lower incidence of postoperative occurrences, the evolution of minimally invasive operations made room for the growth of robotic surgery. Mainly because it is a method that requires high financial investment and time for technical proficiency, the evaluation of its benefits has become necessary in all the procedures in which it is being used^18^ ^,^ ^19^.

Since the surgical approach technique itself is very similar to laparoscopic inguinal repair, robotic-assisted TAPP access was performed smoothly even by surgeons who had no previous experience with robotic surgery^12^ and even by residents^15^. There were only a few changes in the operation in order to ensure the best anchoring of the robot tweezers and the placement and fixing of the screens.

It is worth noting the improvement of the robotic operation in relation to videolaparoscopy: the wristed instruments of the robotic surgery brought greater ease in the manipulation of the tweezers and the techniques of fixation of the mesh, which directly influences the postoperative outcomes^17^. They also present a smaller “leverage effect” due to the poor angulation and positioning of the trocars that occurs in laparoscopic surgery, an innovation that allows the attenuation of tremors and rude movements, guaranteeing greater technical precision and less tissue trauma - an important determinant in the lower incidence of post-chronic pain operative. The dual camera system also allows 3D visualization of the surgical field even more magnified than in laparoscopic surgery. The learning curve of robotic surgery is fast and in a short time it is possible to observe a lower incidence of complications.

 However, both studies that comparatively evaluated the surgical time between robotic repair and laparoscopic or open-ended techniques found greater surgical time in the robotic technique^9^ ^,^ ^10^. Even in one of the studies^9^ that found a discrete increase in the incidence of skin and soft tissue infections in the robot-assisted technique, the author himself suggests a possible correlation between the longer operative time and the higher incidence of infection.

Complications after laparoscopic or open repair are not uniformly described in the literature. The lack of standardization interferes with the variability of the values, which often complicates the comparative analysis. In reviewing the studies with the laparoscopic approach, the most common short-term complications reported in the literature were hematomas and seromas, with a mean incidence of 8% and 7%, respectively. Wound infection occurs rarely at reported rates of approximately 1%^6^. The studies involved in this review present slightly lower rates than those reported in the literature. Comparing the complications between the techniques Eric Charles et al.^9^ reported robotic-assisted, laparoscopic and open techniques with similar values ​​of postoperative occurrences (2.9% in robotic, 3.3% in laparoscopic and 5.2% in open). In another study^7^, considering patients with and without obesity, there was a similar short-term complication rate after robotic vs. open repair. Obese patients who undergo robotic repair have a lower rate of complications after 30 days compared to obese patients submitted to open repair.

The optimal minimally invasive operation in obese patients should provide similar rates of long-term complications that are achieved using minimally invasive approaches in non-obese patients. The results should be similar or better than those achieved with open repair. Lawrence D'Amico et al.^10^ showed that in obese patients the robot assisted approach was comparable and in some cases improved the results compared to those obtained with open procedures. A prospective study using robotic repair in the obese is important to confirm such findings.

 In relation to learning, inguinal hernia repair with TAPP assisted by robot can be performed safely and effectively by residents who participate as surgeons, since there was no difference in the results in paper performed addressing the comparison^15^.

Even though the costs of maintaining the robotic system are close to those of laparoscopic instruments^13^ there is still the additional cost of the robot, which considerably increases the initial investment in the use of this technique. Surgical time, shorter hospitalization time, fewer complications, and improved technical facilities should be counted for long-term cost-benefit assessment. Given the recent increase in the use of robotic technique, this projection has not yet been calculated and in many centers the high cost of robotic material acquisition is still a limiting factor for the use of this form of repair for inguinal hernias.

The main limitation of this review is the retrospective design, presented in six of the eight series, which makes it difficult to analyze pain and quality of life in the postoperative period. It adds to this the fact that there is no information about the uniformity of data collection when performed retrospectively. Another limitation of the studies involved was short-term follow-up, which impaired the evaluation of complications such as chronic pain and paresthesias, which are only evident after months remaining years of the postoperative period.

## CONCLUSION

The advent of robotic surgery represents the future of surgical techniques and should be disseminated and practiced in different centers, even for low complexity surgical diseases, seeking to establish better outcomes and more complete training for the next generation of surgeons. The comparative studies of the literature between the robotic technique and the others did not show superiority in the use of the robot. It is suggested a possible benefit of this technique in obese patients. However, there are no published randomized trials comparing laparoscopic with the robotic inguinal hernia repair, so that future research will need to delineate, in fact, the advantages that one method has over the other.
